# Boc5, a Non-Peptidic Glucagon-Like Peptide-1 Receptor Agonist, Invokes Sustained Glycemic Control and Weight Loss in Diabetic Mice

**DOI:** 10.1371/journal.pone.0002892

**Published:** 2008-08-06

**Authors:** Haoran Su, Min He, Hongmei Li, Qing Liu, Jia Wang, Yiqian Wang, Weiwei Gao, Ling Zhou, Jiayu Liao, Andrew A. Young, Ming-Wei Wang

**Affiliations:** 1 The National Center for Drug Screening, Shanghai, China; 2 The State Key Laboratory of Drug Research, Shanghai Institute of Materia Medica, Chinese Academy of Sciences, Shanghai, China; Monash University, Australia

## Abstract

**Background:**

Our recent discovery of the substituted cyclobutane Boc5, one of the first non-peptidic agonists at glucagon-like peptide-1 receptors, offers the potential of combining oral availability with full agonism capable of eliciting antidiabetic and antiobesity effects. The present study was aimed at determining the *in vivo* pharmacologic properties of Boc5 in both normal and diabetic mice following chronic administration, with emphasis on glycemic control and weight loss.

**Methodology/Principal Findings:**

C57BL/6J and *db/db* mice were treated daily with Boc5 for 4 weeks and a range of pharmacologic parameters, including hemoglobin A1c, intraperitoneal glucose tolerance, insulin tolerance, fasting insulin and leptin levels, food intake, body weight and fat mass, were assessed before and after the treatment. Effects on food intake, gastric emptying, and insulinogenic index were also investigated in animals acutely administered with Boc5. Boc5 (3 mg) was able to induce a durable restoration of glycemic control (normalization of both hemoglobin A1c and intraperitoneal glucose tolerance) in *db/db* mice, following 4 weeks of daily administration. As with peptidic glucagon-like peptide-1 receptor agonists, its glycemic benefit and weight (fat) loss were associated with dose-dependent effects that included reduction in food intake, slowing of gastric emptying (both of which reduce nutrient-drive at β-cells), stimulation of insulin secretion (which was glucose-dependent), and elevation in insulin sensitivity. There was little effect on normal mice treated in the same manner.

**Conclusions/Significance:**

Our findings suggest that Boc5 is the only non-peptidic molecule reported thus far to simultaneously activate this spectrum of antidiabetic effects.

## Introduction

Metabolic syndrome [1], also known as “insulin resistance syndrome” and “syndrome-X”, embraces a clustering of cardiovascular risks that result largely from hypernutrition [2]. The association of some elements, which include obesity, dysglycemia (ranging from impaired glucose tolerance through overt diabetes), dyslipidemia, insulin resistance and hypertension, have been recognized for 40 years [3], [4].

Peptidic glucagon-like peptide-1 (GLP-1) receptor agonists, exemplified by the first incretin mimetic, exenatide, can ameliorate several of these elements, including adiposity [5], dysglycemia, dyslipidemia, insulin resistance and hypertension [6]. They offer the potential to diminish the cardiovascular sequelae of epidemic type 2 diabetes mellitus and obesity, now claiming, respectively, 9.3% [7] and 24% of the U.S. population [7], [8].

From an insulin-centered viewpoint, glycemic control may be augmented three ways: (i) reduction of insulin demand: a decrease in caloric intake, slowing of gastric emptying, reduction in digestion and/or absorption, and diminished endogenous production of glucose (*e.g.* by suppressing inappropriately elevated glucagon secretion) will lead to a reduced need for insulin-mediated nutrient storage; (ii) amplification of insulin secretion, mediated through both glucose-independent and glucose-dependent mechanisms [9]; and (iii) an increase in insulin sensitivity (loss of which is a hallmark of metabolic syndrome). Increases in insulin sensitivity may follow chronic, but not acute, administration of GLP-1 [10] or exendin-4 [11].

Antidiabetic agents rarely cover more than one of the above modes of action. Amylinomimetics excel at reducing glucose appearance (i above), but are devoid of insulinotropic or direct insulin-sensitizing effects (ii and iii above) [12]. Sulfonylureas directly stimulate insulin secretion (ii above), but their glucose-independent action carries a risk for sometimes-fatal hypoglycemia [13]. Glucose-dependent insulinotropic peptide (GIP) receptor agonists powerfully induce glucose-dependent insulin secretion [14], but have no intrinsic effect on food intake, gastric emptying [15], glucagon secretion [16], or insulin sensitivity [17]. Insulin-sensitizing agents, such as the thiazolidinediones (TZD), have antidiabetic efficacy, but do not reduce nutrient assimilation or increase insulin secretion, and typically cause weight gain [18].

The benefits of combination therapy with different antidiabetic agents may derive from apparent advantages of exploring more than one mode of the effects. GLP-1 receptor (GLP-1R) agonists are the only agents thus far known to possess all 3 modes in a single molecule.

All GLP-1R agonists developed to date, or currently under development, are of peptidic nature and require injection. Endogenous GLP-1R agonism can be increased sufficiently for antidiabetic effect by blocking the predominant GLP-1 degrading enzyme, dipeptidyl peptidase-IV (DPP-IV), resulting in an elevation of GLP-1 levels [19]. But this increase in agonism appears insufficient for clinical weight loss [20], and seems to not fully capture the antidiabetic potential of GLP-1R agonism, as exemplified by once-weekly injected exenatide (LAR) [21].

Non-peptidic GLP-1R agonists could, in principal, be orally available and attain such activity. Several screening efforts yielded leads capable of at least partially activating the GLP-1 signaling pathway *in vitro* [22], [23]. But none reported an *in vivo* effect prior to our recent identification of Boc5, a substituted cyclobutane, as a full GLP-1R agonist [24]. The purpose of the present study was to characterize the *in vivo* pharmacologic properties of Boc5, determining its potency and efficacy for antidiabetic and weight loss effects in both non-diabetic and diabetic/obese murine models. In addition, we interrogated a spectrum of GLP-1 actions to examine whether Boc5 fully mimicked the response to peptidic agonists by activating each of the three modes of antidiabetic effects.

In all aspects investigated so far, Boc5 mimics the therapeutic spectrum associated with peptidic agonists, and can fully or partially normalize metabolic derangements manifest in *db/db* mice. Boc5 may thus represent a chemical scaffold for the pharmaceutical development of orally available incretin mimetics.

## Results

### Measures of glycemic control

**Figure 1:**
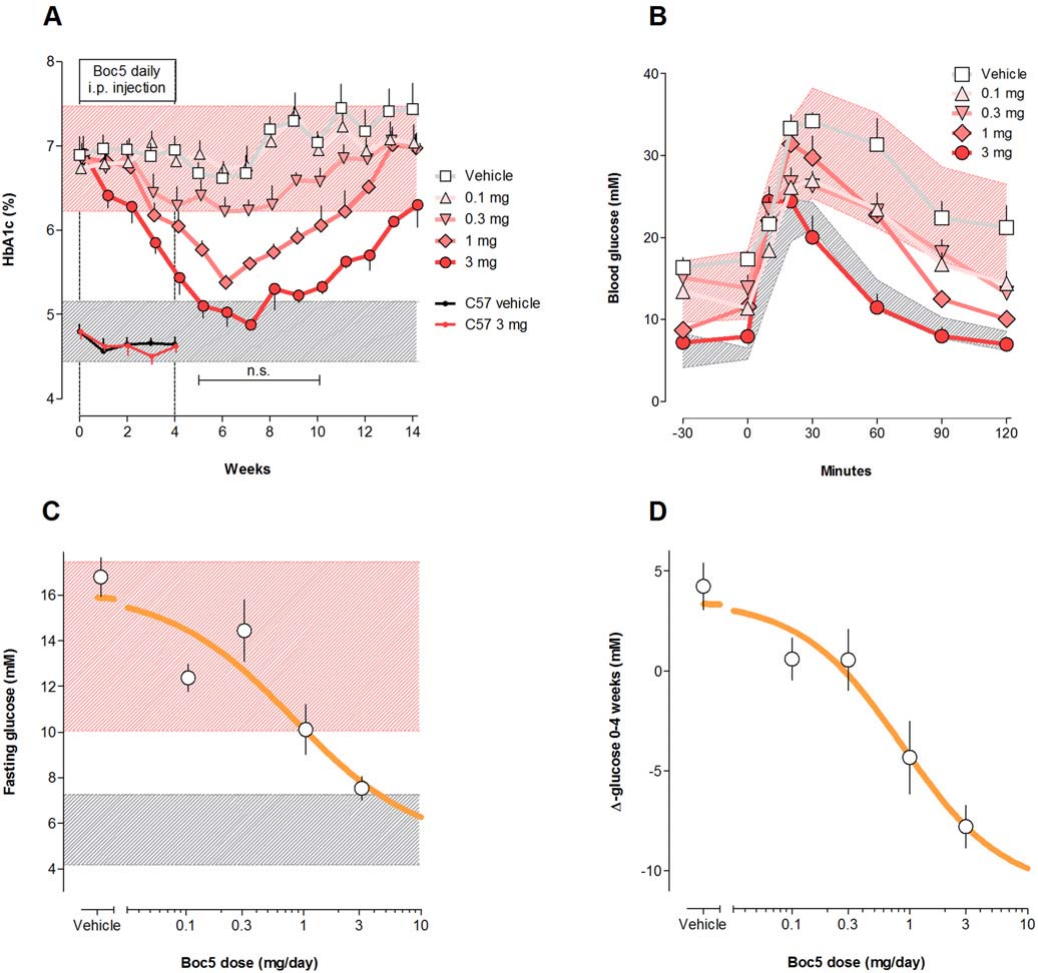
Antidiabetic effects of Boc5. (A) Effect of different doses of daily intraperitoneal (i.p.) Boc5 injection from weeks 0–4 on HbA1c in diabetic *db/db* mice. (B) Blood glucose profiles during an i.p. glucose challenge after 4 weeks of Boc5 treatment in *db/db* mice. The glucose area-under-the-curve integrated from 0–120 min (AUC120) was calculated for each animal before and after Boc5 treatment. (C) Dose response for Boc5 effect on fasting blood glucose after 4 weeks daily i.p. injection. (D) Dose response for changes in fasting blood glucose in individual *db/db* mice over 4 weeks. In all panels the pink and gray bands respectively denote mean±SD (standard deviation) of values measured in *db/db* and non-diabetic C57BL/6J mice (C57) prior to treatment. Symbols are otherwise means±SEM (standard error of the mean). n.s., not statistically significant.

Boc5 administered for 4 weeks did not lower hemoglobin A1c (HbA1c) in non-diabetic C57BL/6J (wildtype) mice. In contrast, in *db/db* mice, daily injection of Boc5 invoked a dose-dependent improvement in glycemic control, as assessed by weekly HbA1c measurement. Reduction in HbA1c was significantly different from control within 1 week of Boc5 treatment (3 mg), and remained so until week 14 (10 weeks after stopping treatment). HbA1c indeed continued to decline after cessation of Boc5 treatment at week 4: it was minimal at week 7, and was maximally different from control at week 9; the ED_50_ for the HbA1c-lowering effect at week 8 was 0.65 mg/day±0.13 log. HbA1c in Boc5-treated *db/db* mice became statistically indistinguishable from levels in non-diabetic C57BL/6J mice within 5 weeks of initiating treatment, and remained so for a further 5 weeks (shown as n.s. in Fig. 1A). Daily oral administration of Boc5 also decreased HbA1c in diabetic mice but it required a high dose (3 mg; Supplemental Fig. S1).

Glucose tolerance was quantified as the area-under-the-curve integrated from 0–120 min (AUC120) after an intraperitoneal glucose tolerance test (IPGTT). Prior to initiating therapy with Boc5, *db/db* mice showed impaired glucose tolerance relative to non-diabetic C57BL/6J control mice (P<0.0001, ANOVA; P<0.01 for each *db/db* dose group *vs* wildtype, Dunnett's multiple comparisons). An IPGTT conducted after 4 weeks of Boc5 treatment revealed a dose-dependent restoration of glucose tolerance, such that the glucose profile of mice receiving 3 mg/day Boc5 was indistinguishable from that of non-diabetic C57BL/6J mice (P = 0.90 for AUC120; ED_50_ 0.31 mg/day; Fig. 1B). The normalization of the glucose profile appeared to reflect reduction of each, fasting glucose, glucose excursion, and rate of decay of blood glucose. Effects on fasting glucose and rate of glucose decay were suggestive of improvements in insulin sensitivity and were examined further.

Treatment with Boc5 had a major effect upon fasting blood glucose in *db/db* mice. Fasting concentrations prior to treatment were ∼12–14 mM in diabetic mice, and in the vehicle-treated controls, worsened somewhat to ∼16 mM over 4 weeks. In contrast, Boc5 treatment dose-dependently (P<0.0001, ANOVA) lowered fasting glucose toward the ∼5–5.7 mM levels observed in non-diabetic C57BL/6J mice (3 mg Boc5 response became indistinguishable from wildtype, Dunnett's multiple comparisons). Thus, in contrast to vehicle treated mice where fasting glucose increased by ∼4 mM over 4 weeks, Boc5 treatment resulted in a reduction of ∼8 mM, essentially normalizing fasting glucose in diabetic *db/db* mice without evidence of hypoglycemia (the lowest individual value was 5.3 mM). The ED_50_ for Boc5-mediated reduction in fasting glucose was 0.82 mg/day (Fig. 1C). The change in fasting glucose from pretreatment until measured after 4 weeks Boc5 treatment was assessed for each of 48 mice for which a data pair was available. The dose response analysis indicated a dose-dependent fall of up to 14.6 mM glucose, and an ED_50_ of 0.87 mg/day (Fig. 1D).

### Measures of adiposity

**Figure 2:**
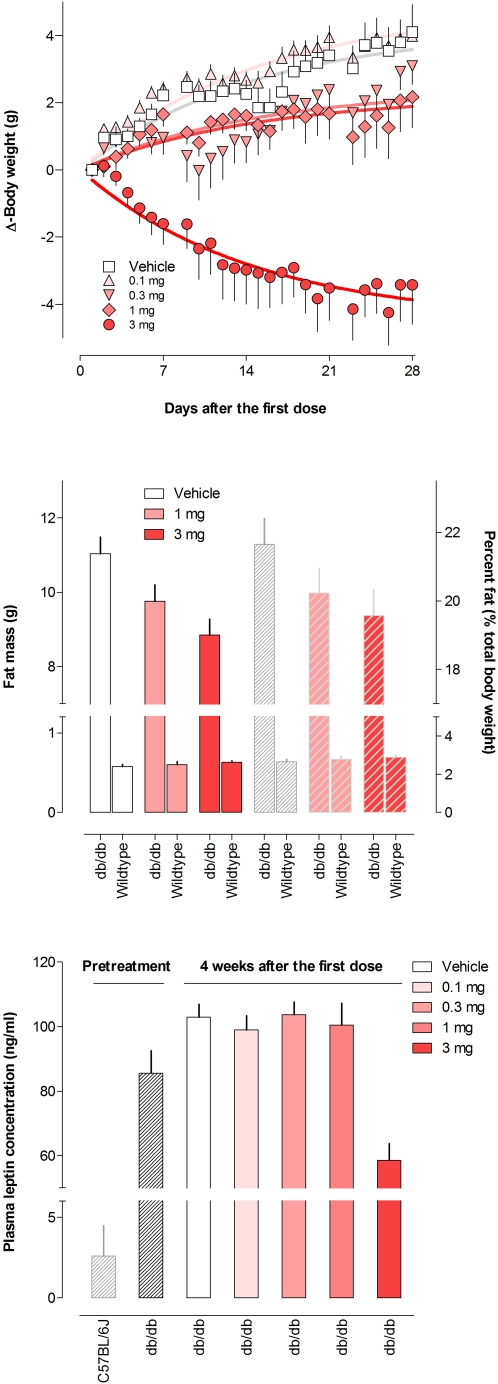
Antiobesity effects of Boc5. (A) Time course of Boc5 effect on changes in body weight. (B) Fat mass and fat percent of body weight. (C) Plasma leptin levels following 4-week daily i.p. treatment with Boc5 in *db/db* mice.

Daily Boc5 administration to *db/db* mice led to a dose-dependent reduction in body weight, relative to the weight gain observed in vehicle treated controls. The ∼7.5 g relative weight loss (3 mg/day dose group) amounted to ∼16% of the initial 46 g body weight of the *db/db* mice (Fig. 2A). Effects on body composition were examined in further experiments on both *db/db* and wildtype C57BL/6J mice treated i.p. for 4 weeks with vehicle, 1 mg, or 3 mg Boc5 daily. Diabetic *db/db* mice were ∼2.2-fold heavier than wildtype mice, and had ∼19-fold more dissectible fat which contributed to an 8-fold elevation of fat as a percent of body weight. The fat depots sampled in these experiments comprised 22% of total body weight in *db/db* mice. This was ∼42% of the value (52±2%) reported for total fat extracted from *db/db* mice by chloroform-methanol [25]. The 2.2 g lost from the 4 fat depots after 3 mg/day Boc5 treatment in the current study may therefore signify a greater amount of total fat loss (*e.g.* 5.3 g if proportionate, representing 71% of the 7.5 g body weight change, relative to controls). While Boc5 invoked weight loss in *db/db* mice, it did not in wildtype mice (P = 0.0014 and P = 0.724, respectively, ANOVA). Similarly, while Boc5 reduced body fat in *db/db* mice, it did not in wildtype mice (P = 0.0067 and P = 0.311, respectively, ANOVA). Although fat as a percent of total body mass trended downward with increasing Boc5 dose in *db/db* mice (P = 0.074, ANOVA), there was no similar trend in wildtype mice (Fig. 2B).

Plasma leptin concentration was measured in *db/db* mice before and after 4 weeks treatment with Boc5. Prior to treatment, leptin was 33-fold higher in *db/db* mice than in wildtype mice (85.6 ng/ml and 2.6 ng/ml, respectively, P<0.0001, unpaired *t* test). Leptin was markedly reduced by Boc5 (P<0.0001, ANOVA), following 3 mg/day administration, to a value of 58.6 ng/ml, that is 68% of the pretreatment and 57% of vehicle-treated levels (P<0.0001, Dunnett's multiple comparisons) (Fig. 2C).

### Measures of nutrient appearance

Of the incretin hormones, only GLP-1 directly limits nutrient appearance, via inhibition on food intake [26] and gastric emptying [15]. GLP-1 may further limit glucose appearance through the suppression of glucagon secretion [27].

**Figure 3:**
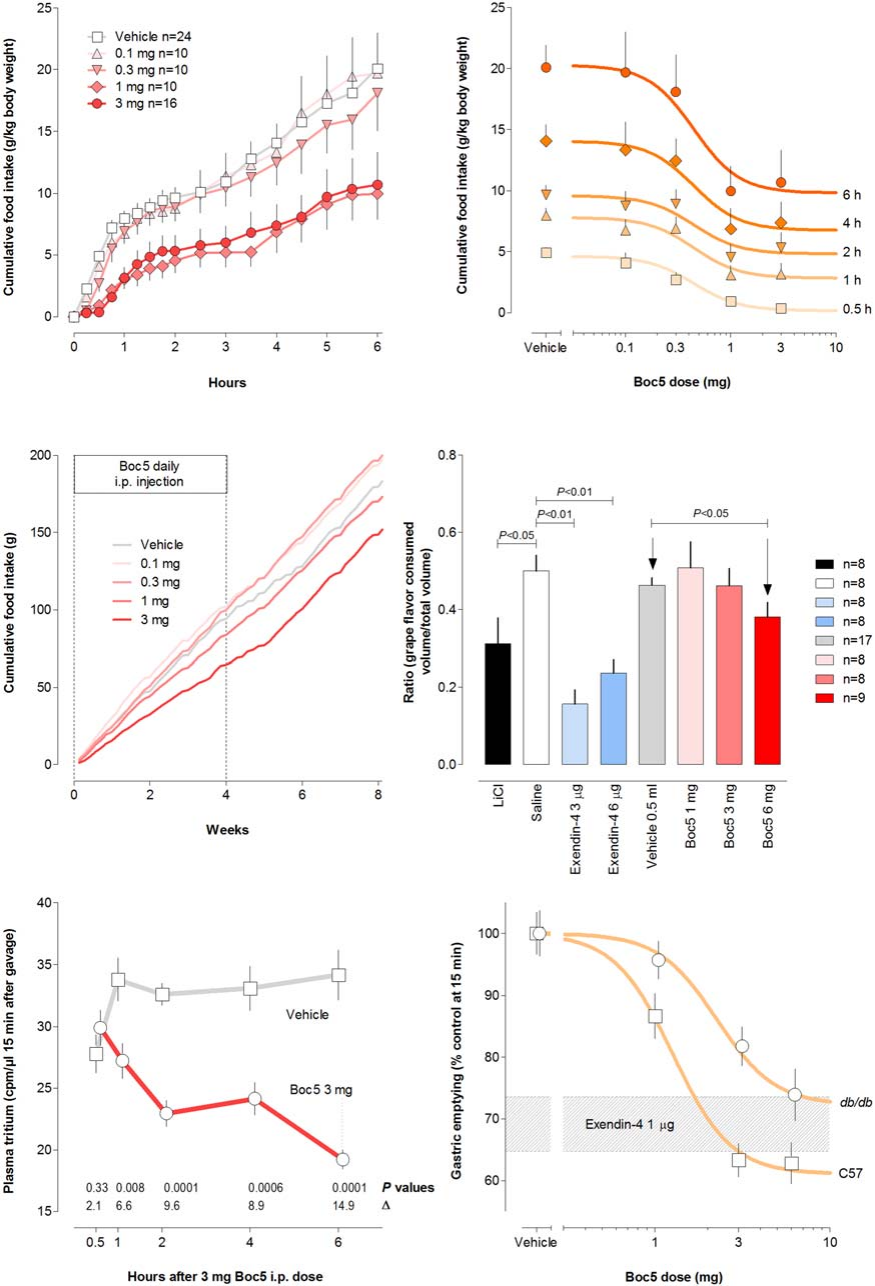
Effects of Boc5 on nutrient appearance, R_a_. (A) Time course of acute effect on food intake in fasted *db/db* mice with different i.p. doses of Boc5. (B) Dose response for effect on food intake at different times after i.p. administration of Boc5. (C) Time course for chronic effect of Boc5 administered for 4 weeks in *db/db* mice on cumulative food intake. (D) Effect of Boc5 on conditioned taste aversion (CTA) in C57BL/6J mice. (E) Effect of Boc5 (3 mg i.p.) on gastric emptying of 3-[^3^H]glucose at different times after Boc5 administration in C57BL/6J mice (C57). (F) Dose response for effect of i.p. Boc5 on gastric emptying measured 6 h after Boc5 administration in both C57 and *db/db* mice. Band is the effect of fully inhibiting (1 µg) dose of exendin-4 in the same protocol (mean±SD).

Boc5 dose-dependently inhibited food intake by up to 50% at 6 h after acute administration in *db/db* mice (P<0.002, ANOVA; ED_50_ 0.91 mg; Figs. 3A and 3B). A similar anorectic effect of Boc5 has been observed in wildtype mice [24]. The absence of Boc5 effect on insulin sensitivity in wildtype mice, reported below, suggests that its anorexic and insulin-sensitizing effects may be dissociable. A similar dose-dependent effect of Boc5 to inhibit food intake in *db/db* mice by up to 42% endured throughout 4 weeks of daily administration, as reflected by cumulative intake (Fig. 3C).

The anorectic effect of Boc5 (1, 3, and 6 mg) was further characterized using a conditioned taste aversion protocol, in which it was compared to exendin-4 (3 and 6 µg). The selection of a particular flavor (*e.g.* grape) was paired with i.p. injection of lithium chloride, considered aversive, which after 2 training runs, reduced total or fractional consumption of the paired flavor *vs* an alternate (*e.g.* cherry). Administration of another nauseogenic agent typically reduces consumption of the paired flavor. Both doses of exendin-4 resulted in a robust conditioned taste aversion (P<0.01, paired *t* test), as has been previously reported [28]. Similarly, the 6 mg (P<0.05, unpaired *t* test), but not the 1 and 3 mg doses of Boc5 invoked significant conditioned taste aversion (Fig. 3D). This study was reproduced in diabetic *db/db* mice in which both lithium chloride (n = 8) and exendin-4 (3 µg, n = 8) elicited significant conditioned taste aversion (P<0.05 and P<0.01, respectively, paired *t* test), while Boc5 (3 mg, n = 8) appeared to be less effective (P = 0.1347, paired *t* test; Supplemental Fig. S2).

Gastric emptying was assessed by the appearance in plasma of tritium derived from 3-[^3^H]glucose 15 min after gavage into fed mice. It has been shown with this label that transport across the stomach wall is negligible, with absorption only occurring after release into the small bowel [29]. Boc5 significantly slowed gastric emptying when administered 1 or more hours before gavage (Fig. 3E). Boc5 i.p. injected 6 h before gavage dose-dependently slowed gastric emptying in both *db/db* and C57BL/6J mice (ED_50_ 2.25 and 1.26 mg, respectively). This effect was equal in magnitude to a fully inhibitory (1 µg) dose of exendin-4 (Fig. 3F). Suppression of gastric emptying by 2 mg Boc5 (ED_80_; i.p. injected 2 h before gavage) could be completed blocked by pretreatment of C57BL/6J mice with 20 µg exendin(9–39), a selective GLP-1R antagonist [30] (data not shown).

### Measures of insulin secretory response

Boc5 has previously been reported to amplify glucose-induced insulin secretion from isolated rat islets [24]. A similar concentration-dependent effect of Boc5 to amplify insulin secretion was observed in rat insulinoma INS-1E cells co-incubated with 16 mM glucose for 30 min. Exendin-4 was equieffective, but 2800-fold more potent in the same assay (data not shown).

**Figure 4:**
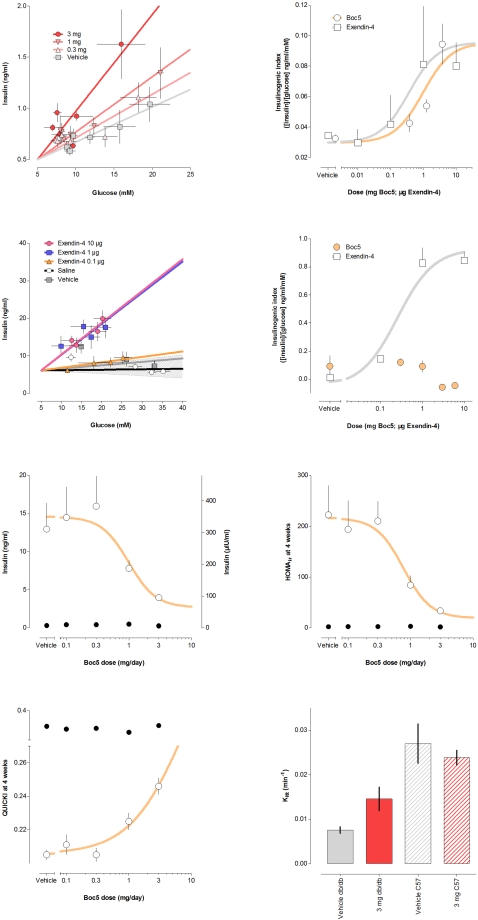
Effects of Boc5 on insulin secretion and insulin sensitivity. (A) Effect of prior administration of Boc5 on insulinogenic index, the slope of the relationship between plasma insulin and blood glucose concentrations in quasi-steady-state, during the decay phase of an intraperitoneal (i.p.) glucose challenge in fasted C57BL/6J (C57) mice. (B) Dose response for effects of Boc5 and exendin-4 on insulinogenic index in C57 mice during an i.p. glucose challenge. (C) Effect of prior administration of exendin-4 and Boc5 on insulinogenic index in fasted *db/db* mice. Gray band depicts the slope range of different Boc5 doses (0.3, 1, 3 and 6 mg). (D) Dose response for effects of Boc5 and exendin-4 on insulinogenic index in *db/db* mice during an i.p. glucose challenge. (E) Dose response for effect of 4-week prior administration of Boc5 on fasting insulin concentrations in *db/db* (open circles) and non-diabetic C57 mice (closed circles). (F) Dose response for effect of Boc5 on HOMA_ir_, an index of insulin resistance derived from fasting insulin and glucose concentrations. (G) Dose response for effect of 4-week prior Boc5 administration on QUICKI, an alternate index of insulin sensitivity derived from fasting insulin and glucose concentrations. (H) Effect of 4-week administration of Boc5 (3 mg/day) on K_itt_, a measure of insulin-mediated glucose clearance, in C57 and *db/db* mice.

Insulin secretory stimulation can be quantified in mice using an insulinogenic index [31], which describes the relationship between insulin secretory response and a secretory stimulus, such as glucose delivered intravenously or intraperitoneally. The slope of the relationship between quasi-steady-state insulin and glucose concentrations (or changes in concentration) quantifies the amplification of secretion typical of incretin action. In the present study, the insulinogenic index derived from an i.p. glucose challenge in the presence of vehicle, was increased 3.2-fold by both Boc5 and exendin-4 (P<0.002, ANOVA; Figs. 4A and 4B). Boc5 was 2700-fold less potent (on a mass basis) than exendin-4 injected i.p. in the same assay (ED_50_ 0.97 mg and 0.36 µg, respectively; Fig. 4B), comparable to the potency ratio observed with INS-1E cells *in vitro*.

A parallel experiment was performed in 12-h fasted diabetic *db/db* mice. While exendin-4 caused a significant and dose-dependent elevation of insulin secretion after an i.p. glucose challenge (ED_50_ 0.25 µg), acutely injected Boc5 at doses up to 6 mg failed to do so (Figs. 4C and 4D). Similar results were observed in 4-h fasted diabetic animals (data not shown).

### Measures of insulin sensitivity *in vivo*

Chronic GLP-1R agonism has been associated with a marked increase in insulin sensitivity in rodents [32]. While the physiologic basis of such an insulin sensitizing effect is still emerging, the effect may be of therapeutic importance, and was assessed here.

Fasting plasma insulin concentration correlates with insulin resistance. It was 43-fold elevated in control *db/db* mice compared to wildtype C57BL/6J mice (12.97 ng/ml and 0.30 ng/ml, respectively, P<0.0002, ANOVA). Boc5, without effect upon fasting plasma insulin concentration in C57BL/6J mice, dose-dependently reduced it by up to 82% in *db/db* mice (P<0.0005, ANOVA; Fig. 4E). HOMA_ir_ is an index of insulin resistance that is derived from fasting glucose and insulin, and correlates inversely in rodents with insulin sensitivity measured by euglycemic clamp [33]. HOMA_ir_ was 102-fold elevated in vehicle-treated *db/db* control mice *vs* C57BL/6J non-diabetic mice. Again, while Boc5 had no consistent effect in C57BL/6J mice, it dose-dependently decreased HOMA_ir_ by up to 91% in *db/db* mice (P<0.0008, ANOVA; Fig. 4F). QUICKI, similarly derived from fasting insulin and glucose, is an index of insulin sensitivity that correlates with clamp-derived measures in mice [34]. It was unaffected by Boc5 in C57BL/6J mice, but was dose-dependently increased by Boc5 in *db/db* mice (P<0.0001, ANOVA; Fig. 4G).

Insulin sensitivity after 4 weeks of treatment was assessed in separate insulin tolerance test experiments by the rate of glucose lowering in response to exogenous insulin. The overall glucose response in mice is typically an initial fall in plasma concentration (attributable to insulin, and subject of this analysis), followed by a rise (attributable to glucagon and other counter-regulatory hormones). The initial rate of glucose fall in response to 2 IU/kg recombinant human insulin (K_itt_) was 3.6-fold higher in vehicle-treated C57BL/6J mice than in *db/db* controls. K_itt_ was unaffected by chronic Boc5 administration in C57BL/6J mice, but was increased 1.9-fold with Boc5 treatment (3 mg/day) in *db/db* mice (P<0.0126, ANOVA; Fig. 4H). Thus, via several independent methods, Boc5 treatment for 4 weeks was associated with an increase in insulin sensitivity in insulin-resistant *db/db* mice, but not in insulin-sensitive C57BL/6J wildtype mice. This result is consistent with the changes in insulin sensitivity observed in rodents following chronic (but not acute) administration of peptidic GLP-1R agonists [35].

## Discussion

The present study examined the *in vivo* pharmacology of the substituted cyclobutane Boc5, the first non-peptidic GLP-1R agonist to show effects in whole animals. In all instances described above, in both the *db/db* mouse model of diabetes/obesity, and in wildtype C57BL/6J mice, Boc5 exhibited actions typical of those observed with peptidic agonists. Where exenatide (synthetic exendin-4) was used as a positive control, maximally-stimulating effects of Boc5 were similar in magnitude to maximally-stimulating effects of exenatide, albeit the latter being ∼3 orders of magnitude more potent.

### Antidiabetic effects

HbA1c is a product of non-enzymatic glycation of hemoglobin. If red cell/hemoglobin turnover is constant, HbA1c as a fraction of total hemoglobin is time-weighted function of recent plasma glucose concentration. The dose-dependent reduction of HbA1c in *db/db* mice shown in Fig. 1A is notable on several counts. First, not only did HbA1c values significantly improve *vs* vehicle-treated controls, but indeed, at some time points and doses, entered the normal range (*i.e*. were “normalized”). This result is consistent with that reported for rodents chronically administered exenatide [11], and is reminiscent of clinical data obtained with once-weekly exenatide, where 86% of patients attained the American Diabetes Association (ADA) treatment goal HbA1c of 7% (*vs* 0% for standard metformin and/or sulfonylurea therapy) [36]. Second, the glycemic benefit endured beyond the period of treatment, and was in fact maximal 2–4 weeks after cessation of therapy. This durable imprint upon the metabolic machinery appears similar to a “memory effect” reported with GLP-1 administration [37].

### Mechanisms underlying glycemic benefit

The blood glucose response to the intraperitoneal glucose challenge shown in Fig. 1B, obtained in *db/db* mice 1 day after the last dose of Boc5, suggests some potential mechanisms of action. The lowering of fasting glucose exhibited there, and in Figs. 1C and 1D alludes to increases in insulin sensitivity. An elevation in insulin sensitivity was further supported by the dose-dependent reduction in fasting insulin concentrations displayed in Fig. 4E. Such a decrease in insulin demand following chronic treatment with Boc5 is in agreement with previous findings in insulin-resistant obese *fa/fa* Zucker rats after 6 weeks of exenatide therapy [32].

Insulin/glucose data pairs enabled the calculation of the HOMA_ir_ and QUICKI indices depicted in Figs. 4F and 4G. Both have recently been demonstrated to correlate with insulin sensitivity in mice, as measured by euglycemic clamp [33]. Prior Boc5 treatment for 4 weeks markedly increased insulin sensitivity, as exemplified by changes in these indices. The increased rate of glucose lowering in response to a fixed (2 IU/kg) dose of insulin, shown in Fig. 4H, provided additional independent evidence for an insulin sensitizing effect of Boc5 in *db/db* (but not insulin-sensitive wildtype) mice.

While GLP-1 agonists have no acute effect upon whole-body insulin sensitivity [38], [39], or in muscle or fat [35], chronic GLP-1R agonism exerts an insulin-sensitizing effect in humans [40] and rodents [10], [35]. The insulin-sensitizing effects of Boc5 observed here is consistent with the response to chronic GLP-1R agonism.

A leptin-mediated feedback that limits lipotoxicity by confining triglyeride to adipocytes [41] may be overwhelmed in hypernutrition. In these circumstances, insulin resistance is associated with ectopic deposition of lipid in non-adipocyte tissues such as muscle, liver and β-cells. An anti-lipogenic potential of GLP-1 is suggested by its inhibition of nutrient assimilation and by an association with higher rates of fat oxidation and energy expenditure in humans [42]. Such an effect in liver is further evidenced by reversal of hepatic steatosis and oxidative stress in *ob/ob* mice following exenatide treatment [43], and is supported in type 2 diabetic patients by amelioration of surrogate markers of nonalcoholic fatty liver disease [44]. The coincidence in the present study of a restoration of insulin sensitivity and of a reduction in leptin concentration (towards a modulable range) would fit with an anti-lipogenic effect of Boc5 to restore triglyceride homeostasis. Cellular evidence for such an action will be the basis of future studies.

The GLP-1 action that first identified it as a drug target was its amplification of glucose-dependent insulin secretion [9]. Even though plasma insulin is typically reduced by exogenous GLP-1R agonists through their several glucose-lowering actions, amplification of insulin secretion can be revealed with indices such as HOMA-B [45] and the insulinogenic index [46]. The effects of Boc5 shown in Figs. 4A and 4B to treble the insulinogenic index directly support an incretin agonist action. The maximal effect of Boc5 was similar to that of exenatide observed in the same experimental protocol. Likewise, the insulin/glucose regression lines of various Boc5 doses intersected with the X-axis (glucose) at ∼5 mM. That is, insulin secretion ceased at plasma glucose concentrations below ∼5 mM, implying preservation with Boc5 of the characteristic over-ride of insulin stimulation during hypoglycemia [47].

An abnormal plasma insulin response to the i.p. glucose challenge was observed in either saline- or vehicle-treated diabetic *db/db* mice (Fig. 4C), consistent with previously findings reported in the literature [48], [49]. Such a reduced capacity for insulin secretion was shown to be associated with a decrease in pancreatic insulin storage [48]. Acute treatment with exenatide, but not Boc5, dose-dependently stimulated insulin secretion (Fig. 4C) with a 7.2-fold increase in the insulinogenic index (Fig. 4D) compared to a 3.2-fold increase in C57BL/6J mice (Fig. 4B). The inability of acutely administered Boc5 to elicit insulin responses under hyperinsulinemia may result from its poor potency as the compound is approximately 2700 times less potent than exenatide (Fig. 4B).

Gastric emptying, only recognized as being of glucoregulatory significance comparatively recently [50], is slowed by several meal-related peptides, including GLP-1, secretin, amylin and cholecystokinin [15]. The data shown in Figs. 3E and 3F affirm an effect of Boc5 to dose-dependently slow gastric emptying in both *db/db* and wildtype mice, with a maximal effect similar to that of exenatide in the same protocol. Like lithium chloride and exenatide, Boc5 at a higher dose (6 mg) induced conditioned taste aversion in C57BL/6J mice (Fig. 3D). This tendency was also observed in *db/db* mice treated with 3 mg Boc5 although the effect was not statistically significant (Supplemental Fig. S2). Such a satiety action exerted by Boc5 provides further evidence that this molecule works as a true GLP-1 mimetic *in vivo*.

### Antiobesity effects

As with peptidic GLP-1R agonists [26], Boc5 administration dose-dependently reduced both acute (Figs. 3A and 3B) and chronic (Figs. 3C) food intake. We surmise that food intake inhibition with Boc5 is important to its dose-dependent reduction (or slowing of gain) in body weight shown in Figs. 2A. For an ideal antiobesity therapy, it is preferred that the weight loss is predominantly from fat. Analysis of carcasses from mice chronically treated with Boc5 indicated that in *db/db* mice, mass was preferentially lost from fat, while in lean wildtype mice treated in the same manner, fat mass was preserved. This was associated with a dose-dependent decrease in fat as a percent of body weight (Fig. 2B). Plasma leptin is a signal derived from fat-replete adipocytes, and is considered a homeostatic effector of body energy content [51]. The marked reduction in *db/db* mice of plasma leptin concentration towards normal with Boc5 treatment (3 mg/day), as shown in Fig. 2C, was consistent with fat loss.

It was notable in the present study, that the Boc5 efficacy for glycemic control (ED_50_ values between 0.3 and 1 mg/day) was generally greater than for weight loss (daily dose of 3 mg). This is in agreement with the preclinical and clinical experience with a number of agents acting upon the GLP-1 axis (incretin mimetics and DPP-IV inhibitors), wherein weight loss is more difficult to achieve than glycemic control [52].

### Pharmaceutic implications

Therefore, in all aspects thus-far investigated, Boc5 is a true mimetic of peptidic GLP-1R agonists. A potential advantage of small molecule organics such as Boc5 over peptidic agonists is the ability to survive the proteolytic environment of the gut, thereby permitting oral availability. This has yet to be fully realized with Boc5 or its analogues, possibly through improvement of their oral bioavailability. Another attractive feature over the already-marketed orally-available DPP-IV inhibitor drugs, demonstrated in the present study, is the attainment of full GLP-1R agonism. DPP-IV inhibitors can invoke clinically meaningful antidiabetic effect through elevation of endogenous GLP-1, and perhaps other regulatory peptides. But they appear to be incapable, even with near-total inhibition of DPP-IV enzymatic activity, of generating sufficient GLP-1 action to invoke weight loss [52]. Boc5, which exhibits both antidiabetic and weight loss effects, may thus represent a starting point for a new class of oral agents targeting metabolic diseases.

## Materials and Methods

### Animals

Eight-week-old C57BL/6J mice of both sexes (22–25 g; Shanghai SLAC Laboratory Animal Co., Shanghai, China) and eight-week-old C57BL/6J-m+/+ Lepr^db^ (*db/db*) mice of both sexes (the Model Animal Research Center of Nanjing University, Nanjing, China) were housed at 22.7±0.8°C in a 12∶12 h light∶dark cycle and were fed and watered *ad libitum*. Animal experimentation was conducted in accordance with regulations approved by the Animal Care and Use Committee, Shanghai Institute of Materia Medica, Chinese Academy of Sciences.

### Chronic *in vivo* studies

*db/db* mice were confirmed as diabetic and assigned into 5 treatment groups (n≥8 per group) with matched HbA1c (Glycosal HbA1c kit, Bio-Rad Laboratories Inc., Hercules, CA and DS1 Glycosal HbA1c Analyzer, Drew Scientific, Barrow in Furness, U.K.), body weight and sexes. A parallel study with the same treatment regimen was carried out in non-diabetic C57BL/6J mice of both sexes (n≥6 per group) for comparison. They were injected i.p. or gavaged once daily with 0 (vehicle control), 0.1, 0.3, 1 or 3 mg Boc5 (1% DMSO, 20% PEG400 in saline, pH 7.4, 0.5 ml) for 4 (i.p. route) or 6 weeks (oral route; n = 7 per group). Blood samples were collected from either the eye socket or the tail vein. HbA1c and overnight fasting blood glucose (using a Freestyle Mini™ blood glucose monitoring system; Abbott Diabetes Care Inc., Alameda, CA) were assayed weekly, and food intake and body weight were measured daily. Before and after treatment, each animal was fasted overnight, measured for plasma insulin levels and challenged i.p. with 2 g/kg D-glucose (Sigma-Aldrich, St. Louis, MO; intraperitoneal glucose tolerance test, IPGTT) followed by serial sampling of blood glucose. A terminal sample was taken for determination of leptin concentrations in treated mice.

In a separate study using an insulin tolerance test (ITT) to assess insulin sensitivity, both *db/db* and C57BL/6J mice were chronically treated with Boc5 for 4 weeks, as described above (n≥6 per group). The insulin tolerance tests, similar to those developed for clinical use [53], comprised a 2 IU/kg i.p. challenge with recombinant human insulin (Humulin® R, Lilly Egypt, Giza, Egypt) followed by glucose sampling at 30 min intervals. The rate of insulin-mediated fall in plasma glucose was quantified in each animal by least-squares fit of consecutive non-rising glucose values to a single-component exponential decay (Prism version 5, GraphPad Software, CA, San Diego) to derive an initial rate, K_itt_.

At the end of the study, mice were sacrificed to dissect and weigh white fat pads (mesenteric, gonadal, retroperitoneal and inguinal) and brown adipose tissue. Weights were summed and expressed as a fraction of total body weight.

### Acute *in vivo* studies

Overnight-fasted *db/db* mice of both sexes were injected i.p. with vehicle or 0.1, 0.3, 1 or 3 mg Boc5 formulated as above (n≥10 per group). Individually caged mice were exposed to a pre-weighed food pellet, which was then reweighed every 15 or 30 min for 6 h to determine cumulative intake. Insulinotropic actions of Boc5 and exendin-4 (Sigma-Aldrich) were measured in 4-h fasting C57BL/6J male and 12-h fasting *db/db* mice 10 min (exendin-4 at doses of 0.01, 0.1, 1 and 10 µg) or 6 h (Boc5 at doses of 0.3, 1, 3 and 10 mg) after i.p. administration (n≥6 per group). Glucose and insulin levels were assessed between 0 and 120 min upon an i.p. glucose challenge (IPGTT) as above.

### Gastric emptying

Rates of emptying were assessed from the appearance in plasma of gavaged labeled glucose, which is not absorbed until it passes the stomach. The method was a derivation of that developed in rats [29]. Pilot studies performed to determine the time course of gastric label release and absorption indicated that blood sampling 15 min after gavage was optimal.

Subsequent studies examined the interval between Boc5 dosing and the assessment of gastric emptying. Conscious and fed male C57BL/6J mice were divided into Boc5 or vehicle treated groups for each time point (n≥12). At 0 h, they were injected i.p. with 3 mg Boc5 formulated as above or vehicle. 3-[^3^H]glucose (1 µCi in 0.5 ml distilled water; GE Healthcare, Piscataway, NJ) was gavaged 0.5, 1, 2, 4, 6 h following the treatment and blood from an orbital bleed was collected 15 min later for measurement of plasma tritium activity using a Microbeta scintillation counter (PerkinElmer, Boston, MA).

Dose-response for the effect of Boc5 treatment was then assessed at the time-point (6 h) where differences from vehicle-treated mice were greatest. Dose-response studies in both C57BL/6J (n = 12) and *db/db* mice (n = 14) used Boc5 doses of 0, 1, 3 or 6 mg administered 6 h before gavage with 3-[^3^H]glucose. Exendin-4 (1 µg in 0.1 ml saline, n = 6), used as a positive control, was injected i.p. 10 min before gavage with 3-[^3^H]glucose.

Finally, the specificity of Boc5 to suppress gastric emptying was examined with a GLP-1R selective antagonist, exendin(9–39) (Ana Spec, San Jose, CA). C57BL/6J mice as above were assigned to vehicle, Boc5 or Boc5 plus exendin(9–39) treatment groups (n≥8). Boc5 (2 mg) or vehicle was given i.p. 2 h and exendin(9–39) (20 µg in 0.1 ml saline) 1 h before gavage with 3-[^3^H]glucose.

### Conditioned taste aversion

Male C57BL/6J mice (7–8 weeks old) or diabetic *db/db* mice (8–10 weeks old) were individually housed and subjected to a training schedule for 5 days, during which they were presented with two water bottles at the same time (9:30–11:00 AM) for 1.5 h each day followed by an i.p. injection of saline (2% of body weight). Animals were randomly grouped (n≥8 per group) based on their body weight at the end of training. On Conditioned Day 1, the water was replaced with two bottles of either cherry or grape Kool-Aid (Kraft Foods North America Inc., Rye Brook, NY) as “Flavor 1” [0.15% saccharin (Sigma-Aldrich) with 0.05% Kool-Aid] for 1.5 h. The following day was a Rest Day, in which animals were only given water for 1.5 h. In the above two days, i.p. injection of 2% body weight of saline was paired with both Flavor 1 and water. On Conditioned Day 2, each mouse was given an alternative Kool-Aid as “Flavor 2” (grape or cherry, respectively) for 1.5 h and was immediately weighed thereafter, injected i.p. with either 2% body weight of saline, 0.15 M LiCl (Sigma-Aldrich), 0.1 ml of exendin-4 (3 µg or 6 µg), 0.5 ml of vehicle solution (1% DMSO and 20% PEG400 in saline) or 0.5 ml of the vehicle solution containing 1 mg, 3 mg or 6 mg Boc5. Another Rest Day was followed and this 4-day conditioned taste course was then repeated once. On the Test Day, mice were simultaneously given both flavors in two bottles for 1.5 h with a position alternation after 45 min, and the fluid intake was measured subsequently. Conditioned taste aversion was signified by either the change in flavor volume or preference ratio [(Flavor 2 consumption/total consumption)×100%] [54].

### Clinical chemistry

Fasting plasma insulin and leptin levels were analyzed using respective ELISA kits (EXRMI-13K and EZML-82K) supplied by Linco Research (St. Charles, MO).

### Data analysis

Dose- and concentration-responses were analyzed using Prism version 5 (GraphPad) to fit 4-parameter sigmoid functions. General effects were tested using 1-way ANOVA. Except where noted otherwise, pair-wise comparisons were performed using Dunnett's test for multiple comparisons, and *t* tests for simple pairs (InStat 3, GraphPad). Data throughout are stated as means±SEM unless otherwise specified. Two-tailed significance was tested at α = 0.05. Where possible, experiments were designed with a sample size calculated from preliminary data to yield a power of B = 0.8.

## Supporting Information

Figure S1 Effect of different doses of daily oral Boc5 administration from weeks 0–6 on HbA1c in diabetic *db/db* mice (n = 7 per dose group).
(0.70 MB TIF)

Figure S2 Effect of Boc5 on conditioned taste aversion (CTA) in diabetic *db/db* mice (n = 8 per dose group)
(0.57 MB TIF)
