# Supplementary figures and images for: Boc5, a Non-Peptidic Glucagon-Like Peptide-1 Receptor Agonist, Invokes Sustained Glycemic Control and Weight Loss in Diabetic Mice
Source: PLoS One. 2008 Aug 6;3(8):e2892. doi: 10.1371/journal.pone.0002892 (PMC2483413; doi:10.1371/journal.pone.0002892)

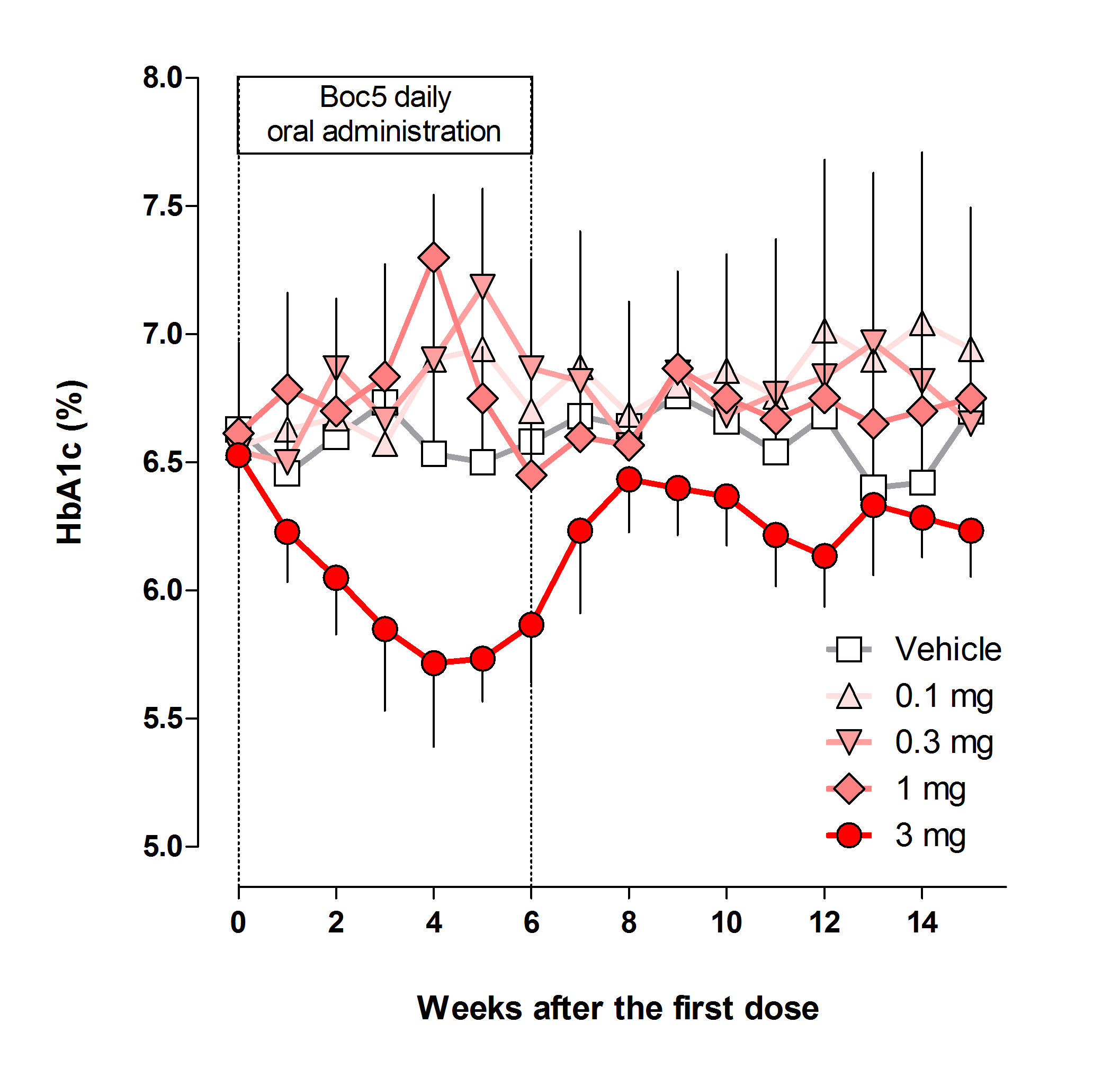

Supplement: Figure S1 — Effect of different doses of daily oral Boc5 administration from weeks 0–6 on HbA1c in diabetic db/db mice (n = 7 per dose group). (0.70 MB TIF) [file pone.0002892.s001.tif]

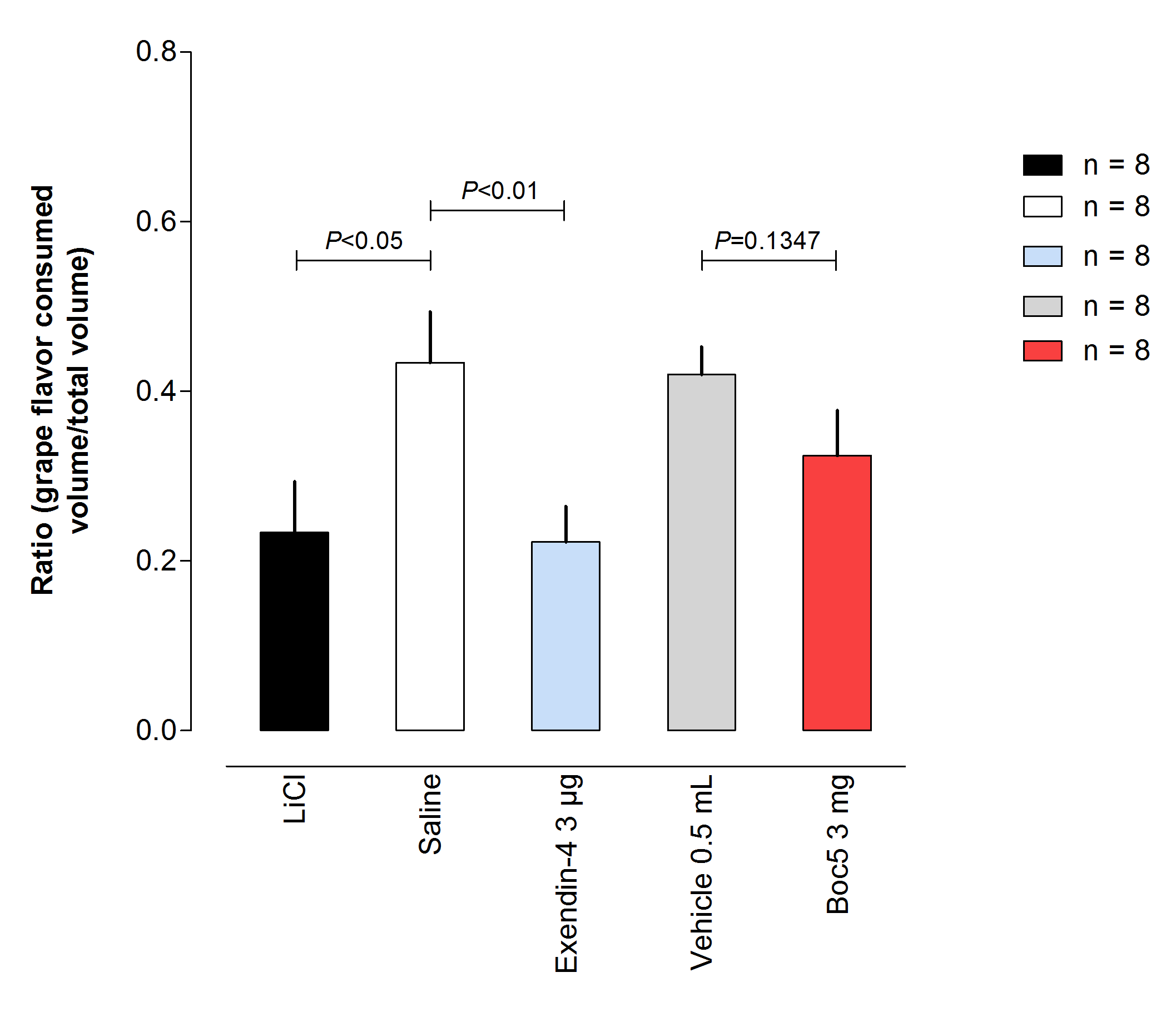

Supplement: Figure S2 — Effect of Boc5 on conditioned taste aversion (CTA) in diabetic db/db mice (n = 8 per dose group) (0.57 MB TIF) [file pone.0002892.s002.tif]
